# Bilateral Wrist Tenosynovitis owing to Acute Conversion of Adult T-Cell Leukemia-Lymphoma in a Patient with Rheumatoid Arthritis

**DOI:** 10.1155/2020/8862599

**Published:** 2020-11-06

**Authors:** Akira Hashimoto, Motoki Sonohata, Satomi Nagamine, Hiromu Yoshizato, Masaaki Mawatari

**Affiliations:** Department of Orthopedic Surgery, Faculty of Medicine, Saga University, Nabeshima 5-1-1, Saga 849-8501, Japan

## Abstract

Human T-cell leukemia virus type 1- (HTLV-1-) associated arthritis is a relatively common disease. However, tenosynovitis owing to adult T-cell leukemia-lymphoma (ATL) is a rare condition. To the best of our knowledge, there have been no reports of tenosynovitis caused by conversion to acute ATL from one of the other ATL types. We present the case of a 60-year-old woman with rheumatoid arthritis (RA) with bilateral wrist tenosynovitis owing to the conversion to acute ATL from one of the other ATL types. She had swelling around the bilateral wrist joint under well controlled RA inflammation. She had no symptoms, physical findings, or laboratory findings indicative of conversion to acute ATL from one of the other ATL types. She underwent tenosynovectomy on the volar and dorsal sides of the left wrist joint to diagnose the cause of swelling around the bilateral wrist joint. Pathological analysis revealed diffuse proliferation of medium-sized atypical CD4(+) lymphocytes. Interestingly, she was diagnosed with wrist tenosynovitis caused by an acute ATL type. This diagnosis suggested that clinicians must consider ATL in connection with atypical wrist tenosynovitis in HTLV-1-endemic areas.

## 1. Introduction

Adult T-cell leukemia-lymphoma (ATL) is a peripheral T-cell malignancy associated with human T-cell leukemia virus type 1 (HTLV-1). ATL is classified into four clinical types: acute, lymphomatous, chronic, and smoldering [1, 2]. A previous epidemiological study reported that HTLV-1 seropositivity is a risk factor for rheumatoid arthritis (RA) in Japan [3]. Although the clinical characteristics of HTLV-1-associated arthritis (HAA) are similar to those of RA [4–6], only two studies have reported cases of multiple tenosynovial nodules owing to ATL [7, 8]. Hence, we present the case of a patient with wrist tenosynovitis caused by an acute ATL type.

The study protocol adhered to the ethical guidelines of the 1975 Declaration of Helsinki, and the study was approved by the institutional review board of our institute. The patient provided informed consent for publication of the case report and the associated images.

## 2. Case Presentation

The patient was a 60-year-old woman who presented to the Rheumatology Department of our hospital in November 2013 with polyarthralgia (right elbow, bilateral wrists, metacarpophalangeal (MP) joint of the right middle and ring fingers, bilateral knees, and bilateral foot joints) and morning stiffness in both hands. She was diagnosed with seropositive RA (rheumatoid factor- (RF-) positive, 44 IU/mL; anticyclic citrullinated peptide antibody-negative, <0.6 IU/mL) according to the 2010 American College of Rheumatology/European League Against Rheumatism [9]. Thereafter, she was referred to the Hematology and Oncology Department of our hospital owing to her medical history, as she was an HTLV-1 carrier. Moreover, she was diagnosed as having smoldering-type ATL, because she had a total leukocyte count of 1300/mm^3^ with 5% abnormal lymphocytes, serum lactase dehydrogenase levels of 224 IU/L (normal range: 120–230 IU/L), serum calcium levels of 9.9 mg/dL (normal range: 8.7–10.3 mg/dL), serum albumin levels of 4.0 mg/dL (normal range: 3.8–5.0 mg/dL), and no tumor lesion [2]. Her RA had been well controlled using salazosulfapyridine (1000 mg/day) and bucillamine (100 mg/day). Although there had been elevation trends in the soluble interleukin-2 receptor levels and the number of abnormal lymphocytes, the patient showed no symptoms, physical findings, or laboratory findings indicative of conversion from a smoldering type to another type of ATL. Therefore, she was carefully monitored by watchful waiting. She had a medical history of breast cancer surgery and dyslipidemia. Her family history included amyotrophic lateral sclerosis (in her mother) and lung cancer and brain tumor (in her father). In March 2018, skin biopsy and pathological analysis did not reveal any evidence of skin rash associated with ATL.

**Figure 1:**
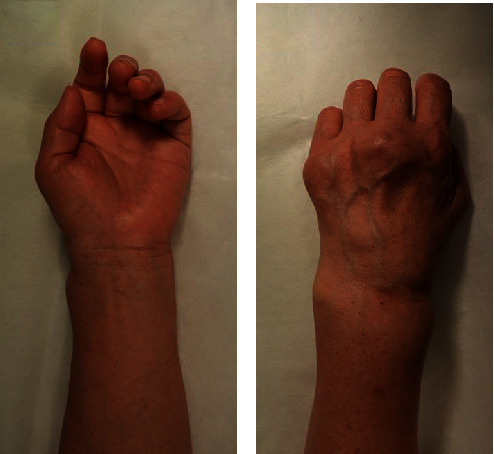
Physical findings of the patient's wrist joint: (a) swelling of the volar side of the patient's left wrist joint and (b) swelling of the dorsal side of the patient's left wrist joint and metacarpophalangeal joints of the left middle and little fingers.

**Figure 2:**
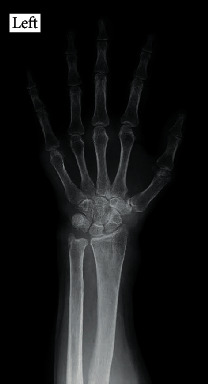
Anterior-posterior plain radiographs showing bone atrophy around the wrist and the metacarpophalangeal joints.

In December 2018, she was referred to the Orthopedic Surgery Department of our hospital for swelling around the left wrist joint since April 2018. She demonstrated swelling on the volar and dorsal sides of her left wrist and on the dorsal side of the MP joints of her left middle and little fingers (Figures 1(a) and 1(b)). She reported experiencing numbness over the area supplied by the left median nerve. The laboratory tests showed the following: total leukocyte count, 2700/mm^3^ with 37.5% abnormal lymphocytes; serum lactase dehydrogenase levels, 269 IU/L; serum calcium levels, 8.9 mg/dL; serum albumin levels, 3.8 mg/dL; C-reactive protein (CRP) level, 0.02 mg/dL (normal range: <0.3 mg/dL); erythrocyte sedimentation rate, 6 mm/h (normal range: 0–17 mm/h); serum matrix metalloprotease-3 (MMP-3) levels, 57.4 ng/mL (normal range: 17.3–59.7 ng/mL); and soluble interleukin-2 receptor levels, 2999 IU/mL (normal range: 127–582 IU/mL). Anterior-posterior plain radiographs of the hands showed bone atrophy around the wrist and MP joint (Figure 2).

**Figure 3:**
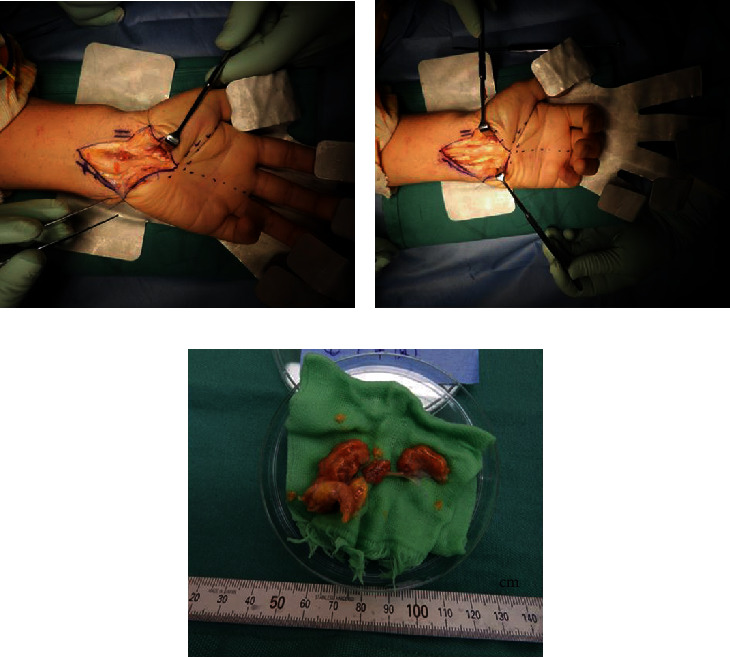
Surgical findings of the volar side: (a) massive tenosynovitis around the left wrist flexors; (b) surgical findings after transverse carpal ligament release, tenosynovectomy, and neurolysis of the median nerve; (c) amount of the volar side resected synovium.

**Figure 4:**
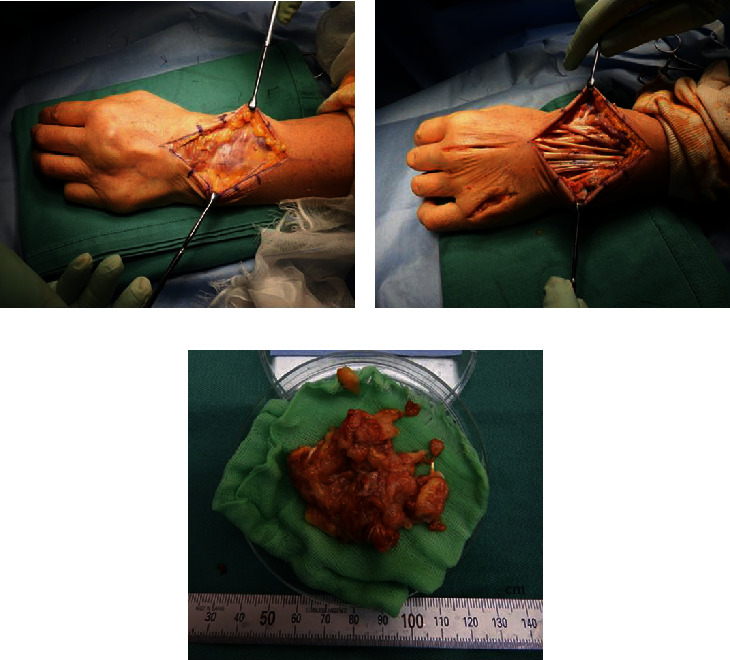
Surgical findings of the dorsal side: (a) massive tenosynovitis around the left wrist extensors; (b) surgical findings after tenosynovectomy and synovectomy of the left metacarpophalangeal joints of the middle and little fingers; (c) amount of the dorsal side resected synovium.

In January 2019, we performed tenosynovectomy on the volar and dorsal sides of the left wrist joint, tenosynovectomy of the wrist and MP joints (middle and little fingers), and neurolysis of the median nerve. Surgical examination of the volar side revealed massive tenosynovitis around the flexors and compression and adhesion of the median nerve owing to tenosynovitis (Figure 3). Surgical examination of the dorsal side revealed thinning of the extensor retinaculum and massive tenosynovitis around the extensors (Figure 4). Moderate synovitis was observed in the MP joint; however, there was mild synovitis in the wrist joint.

**Figure 5:**
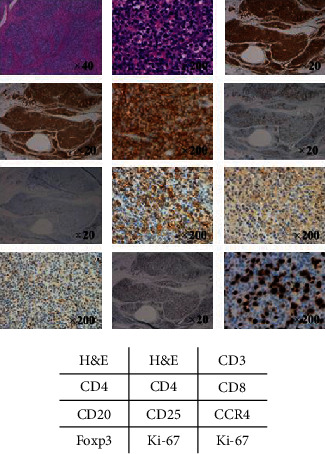
Immunohistological analyses of the tenosynovial tissue biopsy specimens. Abnormal lymphocytes with CD3(+), CD4(+), CD8(-), CD20(-), CD25(+), CD30(-), CCR4(+), and Foxp3(+) profiles are observed. Ki-67 was positively expressed overall. H&E: hematoxylin and eosin; CCR4: CC chemokine receptor 4.

Pathological analysis revealed the diffuse proliferation of medium-sized atypical lymphocytes. The surface marker profile of the atypical lymphocytes in the tenosynovial tissue was as follows: CD3(+), CD4(+), CD8(-), CD20(-), CD25(+), CD30(-), CC chemokine receptor 4(+), and Foxp3(+). The lymphohistiocytic infiltration was positive for Ki-67 (Figure 5). The patient was diagnosed with wrist tenosynovitis caused by an acute ATL type. Subsequently, she underwent multiagent chemotherapies and allogeneic hematopoietic stem cell transplantation. The follow-up period after these treatments was brief; thus, the patient's RA progression after this time remains unknown.

## 3. Discussion

The number of HTLV-1 carriers is approximately 10 million people worldwide, with endemic foci in the Caribbean Basin, Latin America, intertropical Africa, Melanesia, and Japan [10]. HTLV-1 is classified as a complex type C retrovirus belonging to the genus *Deltaretrovirus*, family *Retroviridae*, and subfamily *Orthoretrovirinae* [11]. HTLV-1 is related to ATL, myelopathy, uveitis, Sjogren's syndrome, arthritis, bronchoalveolitis, polymyositis, and RA in Japan [1, 3, 12, 13]. The majority of HTLV-1 carriers remain asymptomatic during their lifetime; however, the lifetime risk of developing ATL among HTLV-1 carriers is approximately 5% for men and 3% for women [14]. Based on the sites of organ infiltration, the presence, absence, and degree of leukemic manifestation, and the high lactase dehydrogenase/calcium levels, the diagnostic criteria of ATL proposed the following four clinical subtypes: the acute, lymphomatous, chronic, and smoldering types [2]. The rates of the acute, lymphomatous, chronic, and smoldering ATL types have been reported to be at 49.5%, 25.7%, 14.2%, and 10.6%, respectively [15]. As the site of organ infiltration of the diagnostic criteria is not clarified concerning the tenosynovium [2], it is necessary to be on alert regarding the symptom of tenosynovitis owing to synovial infiltration of ATL tumor cells. Multiagent chemotherapies with or without subsequent allogeneic hematopoietic stem cell transplantation are recommended for the acute and lymphomatous ATL types [16]. Interferon-*α* treatment combined with zidovudine or watchful waiting is recommended for symptomatic chronic-type and smoldering-type ATL, and watchful waiting alone is recommended for asymptomatic chronic-type and smoldering-type ATL [16]. However, a recent study revealed that the long-term follow-up examination of a patient with favorable chronic- and smoldering-type ATL under only watchful waiting was worse [17]. In addition, interferon-*α* treatment combined with zidovudine was highly effective and significantly improved the survival rates in patients with chronic and smoldering ATL types [18]. Therefore, this patient could have been treated with interferon-*α* combined with zidovudine before acute conversion of the smoldering ATL type.

Past studies have reported a relationship between HTLV-1 and arthritis. In particular, HTLV-1 has been found in the synovial fluid and tissue [19]; HTLV-1-infected synoviocytes produce tumor necrosis factor-alpha (TNF-*α*), which causes synovial proliferation [20]. Moreover, HTLV-1 Tax protein has been detected in the synovial cells and is known to cause synovial proliferation [21, 22]. The clinical characteristics of HAA are similar to those of idiopathic RA and include chronic arthritis and synovial and periarticular inflammation with articular erosion [4–6, 21]. However, HAA is observed in larger joints and shows marked inflammation and relatively mild destruction relative to that observed in typical RA. Anti-TNF agents also exert fewer effects on HAA lesions [13, 23]. A previous study indicated that patients with RA who were HTLV-1-positive had polyarthritis in the large and small joints, while seronegative patients with RA who were HTLV-1-positive had polyarthritis majorly in the large joints [22, 24]. Many studies have reported on HAA [4–6, 19–23]; however, only two reports have referred to tenosynovitis [7, 8]. Our case differs from these two prior reports, as our patient did not have nodule formation caused by ATL-related tenosynovitis, and the cause of tenosynovitis was not the ATL onset but the process of the conversion to acute ATL from smoldering ATL. Massive tenosynovitis was observed around the wrist, although synovitis of the wrist joint was mild, and no elevation of the RF, CRP, and MMP-3 levels was noted during the follow-up period. These findings are not the usual characteristics of RA or HAA, and polyarthralgia involving small joints was noted. Therefore, we assumed that the patient's wrist tenosynovitis was not caused by RA or HAA. Synovial histology performed to obtain a definitive diagnosis revealed that the wrist tenosynovitis was attributed to an acute ATL type.

The patient's independent risk factors for developing ATL were the high HTLV-1 proviral load, older age, family history of ATL, and initial HTLV-1 infection during treatment for other diseases [25]. Interestingly, several studies have reported cases of ATL development in patients with HTLV-1-positive RA who were treated with methotrexate alone, anti-TNF agents, or an anti-interleukin-6 receptor monoclonal antibody [26–28]. Therefore, we should be aware of the possible ATL development in such patients being treated for RA, especially in those with risk factors for ATL development.

In conclusion, ATL is a rare cause of wrist tenosynovitis; however, clinicians must consider it in connection with the atypical wrist tenosynovitis in HTLV-1-endemic areas.

## Data Availability

Data are available on request.
